# Complement anaphylatoxin C5a neuroprotects through regulation of glutamate receptor subunit 2 *in vitro *and *in vivo*

**DOI:** 10.1186/1742-2094-5-5

**Published:** 2008-01-29

**Authors:** Piali Mukherjee, Sunil Thomas, Giulio Maria Pasinetti

**Affiliations:** 1 Department of Psychiatry, Mount Sinai School of Medicine, 1 Gustav L., Levy Place, New York, NY 10029, USA; 2 Department of Neuroscience, Mount Sinai School of Medicine, New York, USA; 3 Geriatric Research and Clinical Center, James J. Peters Veteran Affairs Medical Center, 130 West Kingsbridge Road, Bronx, NY 10468, USA; 4 HRH Prince Alwaleed Bin Talal Bin Abdulaziz Alsaud Institute for Computational Biomedicine, Weill Medical College of Cornell University, New York, NY, USA

## Abstract

**Background:**

The complement system is thought to be involved in the pathogenesis of numerous neurological diseases. We previously reported that pre-treatment of murine cortico-hippocampal neuronal cultures with the complement derived anaphylatoxin C5a, protects against glutamate mediated apoptosis. Our present study with C5a receptor knock out (C5aRKO) mice corroborates that the deficiency of C5a renders C5aRKO mouse more susceptible to apoptotic injury *in vivo*. In this study we explored potential upstream mechanisms involved in C5a mediated neuroprotection *in vivo *and *in vitro*.

**Methods:**

Based on evidence suggesting that reduced expression of glutamate receptor subunit 2 (GluR2) may influence apoptosis in neurons, we studied the effect of human recombinant C5a on GluR2 expression in response to glutamate neurotoxicity. Glutamate analogs were injected into C5aRKO mice or used to treat *in vitro *neuronal culture and GluR2 expression were assessed in respect with cell death.

**Results:**

In C5aRKO mice we found that the neurons are more susceptible to excitotoxicity resulting in apoptotic injury in the absence of the C5a receptor compared to WT control mice. Our results suggest that C5a protects against apoptotic pathways in neurons *in vitro *and *in vivo *through regulation of GluR2 receptor expression.

**Conclusion:**

Complement C5a neuroprotects through regulation of GluR2 receptor subunit.

## Background

The complement system is an essential effector of the humoral and cellular immunity involved in cytolysis and immune inflammatory responses. There is now compelling evidence that complement activation in the brain is a double-edged sword in that it can exert beneficial or detrimental effects depending on the pathophysiological context [1]. Complement has been implicated in diverse human neurodegenerative disorders such as Alzheimer's, Huntington's and Pick's disease [2,3]

The complement system composed of more than 30 proteins is not only activated by antigen-antibody complexes but also by other molecules found in the brain, e.g. myelin and neurofilaments [4,5]. Activation of the complement cascade results in the release of several anaphylatoxins, notable being C3a and C5a, leading to inflammation. C3a and C5a exert their functions by binding to specific receptors, C3aR and C5aR respectively [5].

Functional roles for C3a and C5a have been described during development of cerebellum [6], tissue regeneration [7] and neuronal death [8]. C3a exerts a neuroprotective effect against excitotoxicity-induced death of neurons that are cultured with astrocytes [9]. C5a mediates apoptosis in neuroblastoma cells [8], whereas, it is a potent inhibitor of apoptotic cell death in cultured granule neurons [6].

Neuronal excitation involving the excitatory glutamate receptors is recognized as an important underlying mechanism in neurodegenerative disorders. Excitation resulting from stimulation of the ionotropic glutamate receptors is known to cause neuronal apoptosis. Kainic acid (KA) is an agonist for a subtype of ionotropic glutamate receptor, and administration of KA has been shown to increase production of reactive oxygen species, mitochondrial dysfunction, and apoptosis in neurons in brain [10]. We had earlier reported that the complement component C5 neuroprotects against excitotoxicity; further we showed that mice genetically deficient of complement component C5 revealed a higher susceptibility to KA neurodegeneration [11,12] suggesting that in addition to their pro-inflammatory mechanisms, specific complement components may also mediate neuroprotection. This hypothesis was further supported by evidence showing that C5a may neuroprotect against glutamate mediated apoptosis through the regulation of mitogen activated protein kinase (MAPK) signal transduction pathways [13,14] or by inhibition of caspase-3 activity [15].

Based on the evidence that neuronal death in response to excitotoxic insult involves the regulation of GluR2 receptor expression [16] and that GluR2 receptor expression is reduced coincidental to increase in expression of apoptotic markers like caspase 3 in Alzheimer's brain [17] we decided to explore the role of GluR2 receptors in C5a mediated protection *in vivo *and *in vitro*. In the present study using C5a receptor knockout (C5aRKO) mice we found that neurons were more susceptible to excitotoxicity resulting in apoptotic injury in the absence of the C5a receptor. Our study suggests that C5a may protect against neurodegenerative excitotoxicity and apoptosis in neuronal cells through the regulation of GluR2 receptor expression *in vitro *and *in vivo*.

## Methods

### Primary neuron cultures

Primary cortico-hippocampal cultures of mouse embryonic neurons (gestational day 14–16) were prepared as previously described [15]. Briefly, neurons were seeded at 2 × 10^5 ^cells/well in poly-D-lysine (Sigma) coated 96-well plates or at 10^6 ^cells/well in poly-D-lysine coated 6-well plates and cultured in serum-free chemically defined medium Neurobasal/B27 (2%) supplement and 1% Penicillin-Streptomycin (Gibco-BRL). The absence of astrocytes (<1–2%) was confirmed by the lack of glial fibrillary acidic protein (GFAP) immunostaining verified in parallel studies (data not shown). Northern blot hybridization of total RNA confirmed C5a receptor (C5aR) expression in these primary cortico-hippocampal neurons as previously described [15].

### Human recombinant (hr) C5a and glutamate toxicity

The hrC5a (Sigma) was solubilized in phosphate buffered saline (PBS) and stored at -20°C in disposable 50 μM aliquots; purity was verified by PAGE-Coomassie blue (BRL) as previously described [15]. Chemokinetic potency of hrC5a (EC50: 1.2 × 10^-10 ^M in human neutrophil) was assessed for biological activity (H. Osaka and G.M. Pasinetti, unpublished observations). In our studies, l-glutamate (Sigma) was dissolved in PBS (pH 7.4) and stored at 4°C in 500 mM aliquots, the C5aR antagonist C177 (gift of Dr. Martin Springer, Merck, NJ; 18), was stored in disposable 1 mM aliquots in PBS at -20°C and the MAPK pathway inhibitor PD98059 (Calbiochem) was solubilized in DMSO and stored at -20°C in 10 mM aliquots. Disposable aliquots of DMSO were also stored at -20°C to mimic freeze thaw conditions in vehicle treated cultures. Cultures were treated with glutamate, hrC5a, C177, PD98059 or vehicle (0.001% PBS or 0.01% DMSO, final concentration), as indicated. Glutamate exposure was performed in 7 days old cultures by adding 50 μM glutamate from concentrated stocks into the existing culture media for 24 hr until neuron cultures were collected for viability assays. All cultures and reagents were demonstrated to be free of endotoxin (<10 pg/ml) by Limulus lysate assay (Sigma).

### Kainic Acid (KA) mediated lesions

All procedures involving animals met the guidelines described in the *NIH Guide for the Care and Use of Laboratory Animals *and had been approved by the Animal Facility of the Mount Sinai School of Medicine. Adult male C57B6 mice (25–36 g) (n = 4) were injected intraventricularly with KA (Sigma) to induce hippocampal lesions as previously described [11]. Mice were anesthetized using Avertin (tribromoethanol, 125 mg/kg body weight). KA (80 ng/0.5 μl volume) or vehicle (0.5 μl PBS) was injected unilaterally into wild type (WT) (control mice) (n = 4) and C5aRKO mice (n = 4) in the lateral ventricle using a 5 μl Hamilton syringe attached to stereotaxic apparatus. Mice were sacrificed 72 hr after injection and brains were quickly removed, rinsed in cold PBS and immersed in methylbutane at -25°C for 3 min. 10 micron slices from the frozen brains were mounted on polylysine-coated slides and stored at -70°C.

### Lactic acid dehydrogenase (LDH) Assay

Glutamate neurotoxicity was assessed by measuring the lactate dehydrogenase (LDH) released in culture media 24 hr after glutamate treatment using the Cytotox 96 non-radioactive cytotoxicity assay kit (Promega) (n = 6, experiment repeated three times). Results were quantitated by measuring the wavelength absorbance at 490 nm and were normalized to total LDH in the cells. LDH release was also measured in vehicle treated cultures to control for the effects of DMSO or PBS on cell viability. Data are expressed as percentage of control.

### Hematoxylin and eosin (H&E) staining

Assessment of morphological features of apoptotic damage was done by counting neurons with evident pyknotic condensed nuclei surrounded by cytoplasmic eosinophilia using H&E staining as previously described (12). Primary neuronal cultures plated on chambered slides were fixed in 100% methanol for 10 min., and air-dried. Damaged neurons were quantified *in vitro *from 8–10 randomly selected fields. 10 μm slices from KA and vehicle injected brains of C5aRKO and WT mice were mounted on polylysine-coated slides and damaged neurons were quantified *in vivo *from the different anatomical regions of the hippocampal formation in each brain slice.

### In situ labeling of fragmented DNA (TUNEL assay)

Apoptotic neurons were identified using the terminal deoxynucleotidyl transferase nick end labeling (TUNEL) method of detecting fragmented DNA using the ApopTag kit (Intergen) (n = 4). Briefly, primary cortico-hippocampal cultures on chamber slides were fixed in 100% methanol (15 min) and air-dried following appropriate glutamate and/or hrC5a treatment. The fixed slide cultures were then re-hydrated in PBS and incubated in 0.3% H_2_O_2 _for 5 min. at room temperature. Next, the slides were incubated with TdT enzyme in a humidified chamber at 37°C for 1 hr, followed by 30 min. incubation with anti-Digoxigenin conjugate. TUNEL positive cells were visualized using the chromogen 3-3-Diaminobenzidine (DAB) using the ABC substrate kit (Vector) and mounted for microscopy. Results were quantitated from 8–10 randomly selected fields per well (n = 4 wells).

### Immunocytochemical (ICC) detection of GluR2

For ICC, slides were re-hydrated in phosphate buffered saline (PBS) and incubated in 0.3% H_2_O_2 _for 30 min to block endogenous peroxidase activity. The slides were subsequently incubated with a monoclonal antibody against GluR2 (1:100) (gift of Dr. JH Morrison, Mount Sinai School of Medicine, NY) [19] overnight at 4°C, followed by incubation with secondary antibody (goat anti-mouse HRP; Pharmingen, CA) (1:200) for 60 min. at room temperature. Neurons positive for GluR2 were visualized with DAB using the ABC kit (Vector) (n = 6 slides per group). The slides were then dehydrated in ascending ethanol series, cleared in xylene and mounted with Permount (Sigma) for microscopy. The immunostaining densities were digitized with a high-resolution charge-coupled-device camera (Sony, Tokyo, Japan) and semi-quantified using Bioquant computer-assisted densitometry (Biometrics, Nashville, TN). Camera aperture and focus were adjusted to provide an optimal image. The overall illumination was also adjusted so that the distribution of relative gray values, ie., number of pixels in the image as a function of gray value (0–255), fell within the limits of the system, typically within 30 to 220 gray value units, avoiding a floor or ceiling effect. Once established, the setting remained constant for all the images acquired for all the ICC experiments. Therefore, when all the parameters were fixed, only tissue staining intensities influenced the measured gray value. GluR2 immunoreactive neurons were quantitated from 8–10 randomly selected fields per well (n = 6 wells) and results were expressed as a percent of control.

### Statistical analyses

Difference between groups was assessed by t-test. One way ANOVA was used to compare three or more treatments. Bonferroni's multiple comparison tests was used to detect differences between treatments. For all statistical analyses, the null hypothesis was rejected at *p *< 0.05.

## Results

### C5aRKO mice are more susceptible to KA excitotoxicity and apoptosis than WT littermates

**Figure 1:**
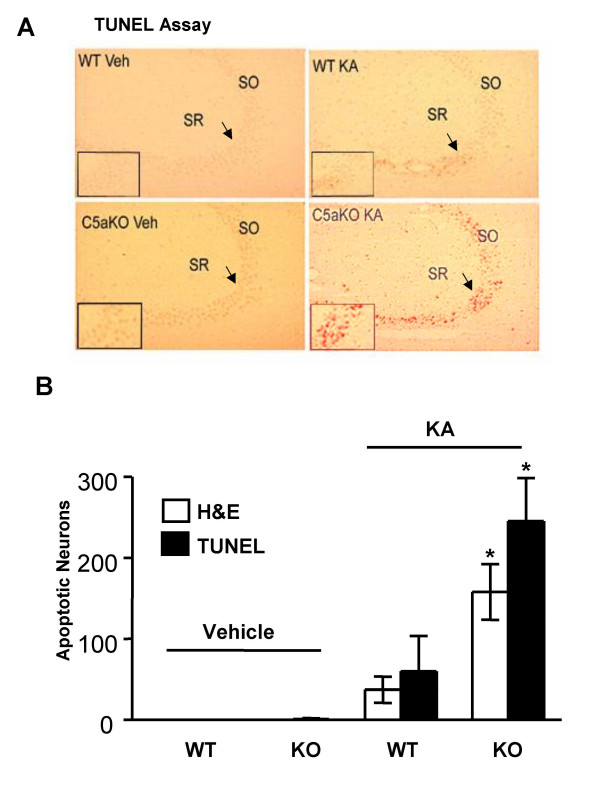
**C5aRKO mice are more susceptible to KA excitotoxicity and apoptosis than wild type littermates**. KA or vehicle (Veh) was administered intraventricularly in C5aRKO mice and age matched wild type litermates (WT) (n = 4). **A**. KA treatment in C5aRKO mice resulted in greater number of TUNEL positive neurons than in KA injected WT littermates (100×). The arrow points to the region in the inset showing the apoptotic neurons. **B**. Both Hematoxylin and eosin (H&E) staining as well as Apoptag (TUNEL) staining methods of assessing apoptosis showed that there were a significantly greater number of apoptotic neurons in C5aRKO mice treated with KA than in WT mice treated with KA in the CA3 hippocampal region. Significance in the number of neurons was assessed using one way ANOVA with Bonferroni's multiple testing correction. All results are expressed as mean ± SD. Hematoxylin/eosin WT and C5aRKO – KA induced: *p < 0.01; TUNEL WT and C5aRKO – KA induced: *p < 0.006. SO = stratum oriens; SR = stratum radiatum.

The excitatory neurotransmitter glutamate is known to play an important role in the induction of excitotoxic neurodegeneration through activation of its receptors. Kainic acid (KA) is a potent glutamate receptor agonist with selectivity towards non-NMDA type glutamate receptors. Both apoptotic and necrotic death of neurons are associated with KA-induced excitotoxicity, suggesting the existence of multiple death pathways induced by glutamate receptor neurotoxicity. Histology performed with hematoxylin/eosin on brain slices from C5aRKO and WT C57B6 mice suggested that C5aRKO mice are more susceptible to KA induced neurodegeneration than age and gender matched WT mice. Furthermore, KA lesions in C5aRKO mice resulted in greater number of TUNEL positive neurons than in KA injected WT littermates (Fig. 1) (ANOVA: Hematoxylin/eosin WT and C5aRKO – KA induced: *p *< 0.01; TUNEL WT and C5aRKO – KA induced: *p *< 0.006). This result suggests that C5aR exerts a neuroprotective role against KA-induced neurotoxicity.

### C5aRKO mice show decreased GluR2 expression *in vivo *in response to KA compared to WT littermates

**Figure 2:**
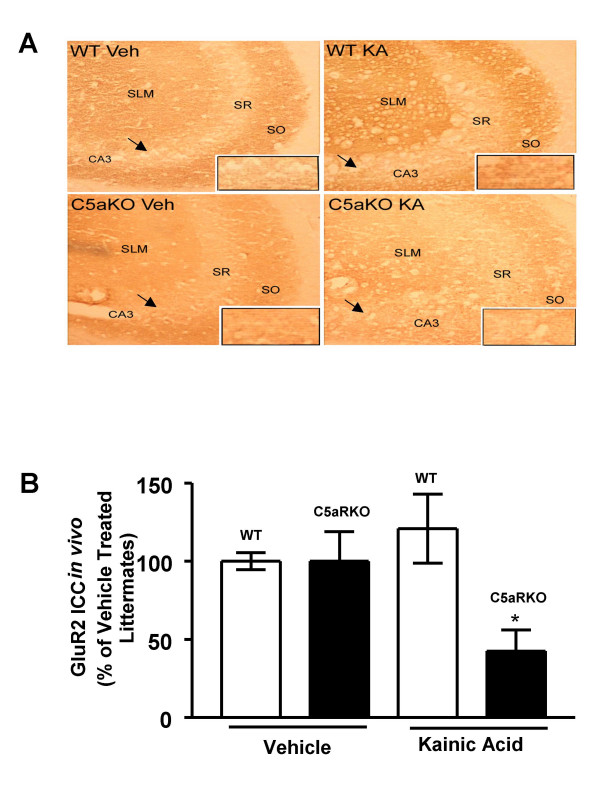
**The hippocampus of C5aRKO mice show decreased GluR2 expression in vivo in response to KA when compared to WT littermates**. KA or vehicle (Veh) was administered intraventricularly in C5aRKO mice and age matched wild type litermates (WT) (n = 4). Frozen hippocampal brain sections from these mice were then probed immunocytochemically for GluR2 expression using GluR2 antibody. GluR2 immunoreactivity was quantitated from 8–10 randomly selected fields and results were expressed as a percent of vehicle treated WT controls. KA significantly decreased the expression of GluR2 immunoreactivity in the mossy fiber region of C5aRKO mice (Fig. A, B). The arrow points towards the region in the inset which shows the GluR2 immunoreactivity in the mossy fibers of the CA3 region. Hippocampal GluR2 immunoreactivity was reduced in C5aRKO mice treated with KA compared to C5aRKO mice treated with vehicle. All results are expressed as mean ± SD. Difference between groups was assessed by t-test, *p < 0.05. SO = stratum oriens; SR = stratum radiatum; SLM = stratum lacunosum moleculare.

Non-NMDA receptor ion channels that can be gated by KA are formed by the glutamate receptor subunits GluR1-GluR4. GluR2 is downregulated in neurons following a wide range of neurological insults [33] and our subsequent study focused on the role of KA on GluR2 in C5aRKO and its WT control. When KA was administered intraventricularly in C5aRKO and wild type mice, we found that the immunostaining of GluR2 receptor protein expression was decreased in C5aRKO mice in response to KA lesions as assessed by immunocytochemical analysis (Fig. 2). Quantitation of GluR2 immunoreactivity after treatment with KA showed a significant decrease in expression of GluR2 in the C5aRKO mice compared to the vehicle treated littermates (t-test: C5aRKO vehicle vs KA induced: *p *< 0.05). The results suggest that C5aR plays a neuroprotective role by upregulating GluR2 against KA-induced neurotoxicity possibly by making the cells impermeable to Ca^2+ ^influx.

### Glutamate induces a receptor specific decrease in neuronal GluR2 expression *in vitro*

**Figure 3:**
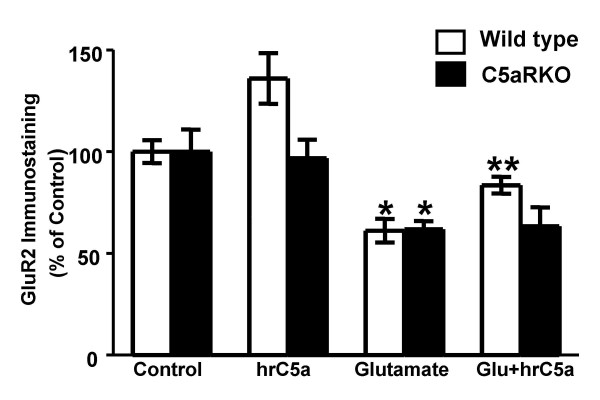
**hrC5a protected against glutamate mediated decrease in GluR2 receptor expression *in vitro***. In primary murine neuronal cultures, 50 μM glutamate for 24 hr significantly decreased GluR2 expression as assessed by GluR2 immunostaining in primary cultures from both WT and C5aRKO mice (n = 4). However, while WT primary cultures treated with glutamate and hrC5a (100 nM) which is able to neuroprotect significantly against glutamate neurotoxicity in WT cultures did not show significant decrease in GluR2, primary cultures from C5aR KO mice co-treated with glutamate and hrC5a showed significant decrease in the expression of GluR2. Results were expressed as a percent of controls and significance was assessed using one way ANOVA with Bonferroni's multiple testing correction. All results are expressed as mean ± SD. WT Control vs Glutamate: *p *< 0.01; WT Control vs Glutamate+hrC5a: **p < 0.0001; C5aRKO Control vs Glutamate: *p < 0.006; C5aRKO Control vs Glutamate+hrC5a: *p < 0.006.

Previous experiments performed to characterize glutamate excitotoxicity in primary cortico-hippocampal neuronal cultures showed that concentrations of glutamate ranging from 25–100 μM cause neuronal toxicity after 12–24 hr in approximately 30–75% cells in *in vitro *cell culture experiments [15]. Subsequent experiments were performed using a dose of 50 μM glutamate for 24 hr, which achieved a significant amount of neuronal death within the linear range of increasing glutamate toxicity assessed by LDH assay in our culture. Moreover, treatment of neurons with the non-competitive NMDA receptor antagonist MK801 (10 μM) blocked glutamate toxicity (not shown). WT primary cultures treated with glutamate and hrC5a (100 nM) which is able to neuroprotect significantly against glutamate neurotoxicity in WT cultures did not show significant decrease in GluR2, whereas, primary cultures from C5aR KO mice co-treated with glutamate and hrC5a showed significant decrease in the expression of GluR2 (Fig. 3) (ANOVA: WT Control vs Glutamate *p *< 0.01; WT Control vs Glutamate+hrC5a *p *< 0.0001; C5aRKO Control vs Glutamate *p *< 0.006; C5aRKO Control vs Glutamate+hrC5a *p *< 0.006). The result suggests that addition of hrC5a to C5aRKO mice neurons do not decrease glutamate toxicity due to the absence of specific C5a receptors.

### C5a protection against glutamate mediated GluR2 depletion *in vitro *is specific for both C5a and glutamate receptors

**Figure 4:**
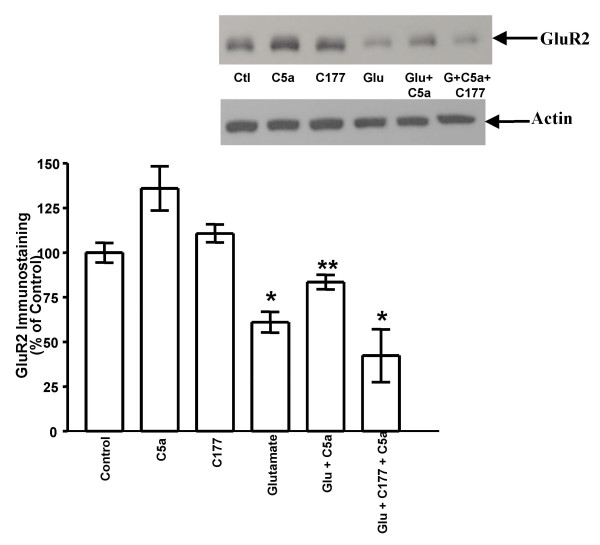
**C5a mediated protection against glutamate mediated GluR2 depletion *in vitro *is specific for both C5a and glutamate receptors**. The hrC5a mediated protection of GluR2 in the presence of 50 μM glutamate for 24 hrs in primary neuronal cultures from WT mice was absent when co-treated with C177. All results are expressed as mean ± SD. WT Control vs Glutamate: **p < 0.0001; WT Control vs Glutamate+C5a: *p < 0.01; WT Glutamate vs Glutamate+C5a: *p < 0.05; Control vs Glutamate+C177+C5a: *p < 0.03. The western blot shows the GluR2 expression in the same experiment.

Previous studies had determined that an optimal dose of 100 nM hrC5a was required to achieve a maximal neuroprotection when pretreated 24 hr before glutamate exposure in primary murine cortico-hippocampal neurons [14,15]. In our final experiment (repeated thrice) designed to determine the specificity of both C5aR and glutamate receptor we treated WT neurons with the antagonists of C5a. C5a alone enhanced the expression of GluR2 and the C5a antagonist C177 *per se *had no effect on GluR2 expression. Glutamate alone had a negative effect on GluR2, whereas hrC5a in the presence of glutamate had a positive effect, to the extent that there was an increase in expression of GluR2. However, the hrC5a mediated protection of GluR2 (Fig. 4) in the presence of glutamate in primary neuronal cultures from WT mice was absent when co-treated with C177 (ANOVA: WT Control vs Glutamate *p *< 0.0001; WT Control vs Glutamate+C5a *p *< 0.01; WT Glutamate vs Glutamate+C5a *p *< 0.05; Control vs Glutamate+C177+C5a *p *< 0.03).

## Discussion

Complement has long been hypothesized to play a role in neuroinflammation and C5a has been postulated to have several different roles in central nervous system disease [20]. Though it has been suggested that C5a-C5aR interaction may lead to increased neuronal cell death in Alzheimer's disease [8] our group has demonstrated a novel protective role for C5a in inflammation models [14,15]. Reiman et al. [21] developed a transgenic mouse that produces C5a exclusively in the brain using the astrocyte-specific, murine glial fibrillary acidic protein (GFAP) promoter. C5a/GFAP mice develop normally and do not demonstrate any signs of spontaneous inflammation or neurodegeneration with age. C5a also plays an important protective role in allergic lung disease by suppressing inflammatory responses and Th2 effector functions as observed in an experimental model of asthma [22]. The C5aRKO mice also develop normally and do not demonstrate any signs of inflammation or neurodegeneration. In this study we observed that C5aRKO mice are more susceptible to apoptosis induced by KA (an analog of glutamate) stimulation compared to the WT littermates.

Neuronal injury mediated by overstimulation of receptors for the major excitatory transmitter, glutamate (Glu), has been implicated in a variety of neurodegenerative conditions [23]. Exposure to neurotoxic concentrations of Glu leads to necrosis *via *the NMDA receptors [24], while overstimulation of the non-NMDA receptors, KA and AMPA, commonly produces a pattern of cell death characteristic of apoptosis [25,26]. Activation of the NMDA receptor stimulates JNK and p38 MAP kinases in cultured cerebellar granule cells (CGCs) [27], and in hippocampal neurons AMPA and KA receptors stimulate ERKs, JNK and p38 kinases [14]. KA and/or AMPA receptor stimulation results in the marked activation of the ERK kinases in oligodendrocytes [28] and striatal slices [29]. There is also evidence that C5a inhibits the spontaneous apoptosis of neutrophils, extending their lifespan after recruitment to sites of inflammation [30]. Other inflammatory mediators including perforin may facilitate this protective function of C5a [31]. We had earlier shown that C5a may protect against glutamate-induced apoptosis in neurons through MAPK-mediated regulation of caspase cascades [14]. In this study we found that C5a may protect against apoptotic pathways in neurons *in vitro *and *in vivo *in part through regulation of GluR2 receptor expression in the brain.

The relative presence of the GluR2 subunit determines the functional properties of AMPA receptors, the mediators of fast excitatory neurotransmission, play a crucial role in synaptogenesis and formation of neuronal circuitry, as well as in synaptic plasticity. Studies show the levels of GluR2 mRNA and protein in CA3 and/or CA1 are downregulated after KA-induced seizures [16,32], indicating that KA-induced reduction of GluR2 is related to GluR2 gene transcription. The mechanism of GluR2 downregulation during KA exposure is still not established. Jia et al. [33] reported that suppression of GluR2 gene promoter activity is associated with KA induced downregulation of GluR2 subunit levels in primary cultured cortical neurons. In our studies we observed that when KA was administered in C5aRKO, there was a reduction of GluR2 receptor protein expression. We hypothesize that during KA induced sensitization in the absence of C5aR, GluR2 is downregulated leading to apoptosis. Animal models of global ischemia and induction of status epilepticus have demonstrated such a downregulation of GluR2 mRNA and protein expression in the CA1 and CA3 regions of rat hippocampus [34,35].

It is widely known that C5a functions by binding to its specific receptor-C5aR. Our results show that exogenous addition of hrC5a alone increases GluR2 protein expression in WT neurons, whereas there was no increase in GluR2 protein levels in C5aRKO mouse neuron. Glutamate alone was toxic for both WT and C5aRKO mouse neuron, but the level of toxicity was lower in WT mouse neuron in the presence of hrC5a since it could access specific C5a receptors. To prove that C5a protection against glutamate mediated GluR2 depletion was specific for both C5a and glutamate receptors we used the C5a antagonist-C177. Our results show that the complement C5a neuroprotects through regulation of GluR2, in addition to mitogen activated protein kinase (MAPK) signal transduction pathways [13,14], and inhibition of caspase-3 activity [15].

## Conclusion

In conclusion we found that the complement C5a protects against apoptotic pathways in neurons *in vitro *and *in vivo *through regulation of GluR2 receptor subunit.

## List of abbreviations

GluR2: Glutamate Receptor 2; KA: Kainic Acid; C5aRKO: C5a Receptor Knock Out.

## Competing interests

The author(s) declare that they have no competing interests.
